# Special Issue “Smart Polymeric Films and Coatings for Food Packaging Applications”

**DOI:** 10.3390/polym15234522

**Published:** 2023-11-24

**Authors:** Sneh Punia Bangar, Yuthana Phimolsiripol, Monica Trif

**Affiliations:** 1 Department of Food, Nutrition and Packaging Sciences, Clemson University, Clemson, SC 29634, USA; snehpunia69@gmail.com; 2 Faculty of Agro-Industry, Chiang Mai University, Chiang Mai 50100, Thailand; yuthana.p@cmu.ac.th; 3 Food Research Department, Centre for Innovative Process Engineering (CENTIV) GmbH, 28816 Stuhr, Germany

Smart polymeric films and coatings represent a significant step forward in packaging technology. Smart polymeric films and coatings for food packaging refer to materials that offer enhanced functionalities beyond basic packaging requirements. Smart polymeric films and coatings play a crucial role in enhancing the shelf life and safety of food products [1,2]. They can provide a protective barrier against external factors such as moisture, oxygen, light, and contaminants. Additionally, they can offer functionalities such as antimicrobial properties, oxygen scavenging, and indicators for freshness. It is very important to consider factors such as food compatibility, regulatory compliance, cost-effectiveness, and sustainability when selecting smart polymeric films and coatings for specific food packaging applications. Additionally, research and development in this field continue to drive innovation, leading to more advanced and efficient packaging solutions. 

Active antimicrobial films and coatings incorporate antimicrobial agents like silver nanoparticles, essential oils, or organic acids to inhibit the growth of bacteria, molds, and yeasts, thereby extending the shelf life of packaged food [3,4].

Oxygen scavenging films and coatings contain oxygen scavengers that absorb and remove oxygen from the packaging, which helps to slow down the oxidation process and extends the freshness of oxygen-sensitive food products [5].

Ethylene-absorbing films and coatings are designed to absorb ethylene gas, a natural plant hormone that accelerates the ripening process in fruits and vegetables. Slowing down the ethylene production can extend the shelf life of fresh produce [6].

Moisture-regulating films and coatings with moisture-absorbing or moisture-releasing properties are intended to maintain an optimal humidity level within the packaging, preserving the quality of the food. pH-responsive films change their properties (e.g., permeability) in response to changes in pH levels, which is particularly useful for products that are sensitive to pH variations [7].

Intelligent packaging with sensors, such as films that incorporate sensors, RFID tags, or QR codes, monitor parameters like temperature, humidity, gas concentration, and freshness indicators. These sensors can provide real-time information about the condition of the packaged food. Nanoemulsion-based films and coatings contain nanoemulsions, which are stable oil-in-water or water-in-oil emulsions, to encapsulate bioactive compounds, enhance antimicrobial properties, or improve the barrier properties of the film. Gas-permeable films and coatings with controlled gas permeability allow for the exchange of specific gases (e.g., oxygen, carbon dioxide and ethylene), which is crucial for the packaging of products like fresh produce and modified atmosphere packaging (MAP) [8]. Time–temperature indicators (TTIs) are films and coatings with indicators that change color or provide other visual cues based on temperature and time, providing consumers with an indication of the product’s freshness and safety [9].

Nanocomposite films and coatings incorporate nanoparticles (e.g., clay, graphene) to improve their mechanical strength, barrier properties, and other functionalities [10].

UV-blocking films and coatings are designed to block ultraviolet (UV) light, which can help protect food products from light-induced degradation and extend their shelf life. Smart coatings can include features like tamper-evident seals or authentication markers to ensure the integrity of the packaged product and protect against counterfeiting [11,12].

These examples represent a range of innovative technologies in the field of smart polymeric films and coatings for food packaging. Each of these technologies addresses specific challenges in food packaging, aiming to improve food’s safety, quality, and shelf life. The availability and commercial viability of these technologies may vary based on regional regulations and market demand. Compliance with food safety and regulatory standards is crucial to the adoption of edible films in the industry.

Edible films and coatings, often referred to as “smart films” when they incorporate advanced functionalities, are innovative materials used in the food industry for various purposes. These films are typically made from food-grade materials like proteins (e.g., gelatin, whey, soy), polysaccharides (e.g., starch, cellulose, alginate), lipids (e.g., waxes, fatty acids), and other natural compounds [2,4,8,10,13,14,15]. They may also contain additives like plasticizers, antioxidants, antimicrobial agents, flavorings, and colorants. These materials offer opportunities for unique and innovative packaging designs that can set products apart on the market, enhancing brand recognition and consumer appeal, with customizable textures, colors, and patterns that enhance the product’s branding and attractiveness to consumers. As consumer preferences and industry standards evolve, smart polymeric films and coatings can be engineered to meet new demands, making them a versatile and future-proof solution [11]. Smart packaging can also provide convenience features for consumers, such as easy-open seals, and portion-controlled dispensers. Smart polymeric films and coatings represent an exciting advancement in the packaging industry, offering numerous benefits for both consumers and producers. They have the potential to revolutionize the way food is packaged and consumed, making it safer, more efficient, and environmentally friendly [12]. By preventing spoilage and preserving freshness, these films and coatings can help to reduce food waste, and can contribute to sustainability efforts through better preservation, as well as by potentially replacing traditional single-use plastics with more environmentally friendly options.

While the initial investment in smart packaging technology may be higher, the benefits in terms of extended product shelf life, reduced waste, and improved consumer satisfaction can lead to long-term cost savings [16]. Again, the specific formulation and application of these smart polymeric films and coatings can vary depending on factors such as the type of food being packaged, storage conditions, and regulatory requirements.
